# Update zur Pathologie der Peniskarzinome

**DOI:** 10.1007/s00120-025-02701-7

**Published:** 2025-10-22

**Authors:** Sigrid Regauer

**Affiliations:** Diagnostic- and Research Institute of Pathology, Referenzzentrum für anogenitale Erkrankungen, Verein interdisziplinäre Interessensgemeinschaft Vulvaerkrankungen (ZVR 174112632), Medical University Graz, MedCampus, Neue Stiftingtalstraße 6, 8010 Graz, Österreich

**Keywords:** Humane Papillomaviren, Lichen planus, Lichen sclerosus, Intraepitheliale Neoplasie, Squamöse intraepitheliale Läsion, Human papilloma virus, Lichen planus, Lichen sclerosus, Intraepithelial Neoplasia, Squamous intraepithelial lesion

## Abstract

Die Inzidenz von Peniskarzinomen beträgt in Europa < 1 %. Etwa die Hälfte der Peniskarzinome entsteht über die Präkanzerose hochgradige squamöse intraepitheliale Läsion (HSIL) durch eine transformierende Infektion mit humanen Papillomaviren (HPV) – am häufigsten High-risk-HPV 16. Die HP-E6/E7-Onkogenproteine binden an Proteine der p53 und Retinoblastomsignalwege. Diese Zellzyklusstörung führt zur zellulären Ansammlung/Überexpression von p16, was als Biomarker für HPV-assoziierte Karzinogenese gilt. Die Mehrheit der HPV-unabhängigen Peniskarzinome entsteht in Läsionen chronischer lichenoider Dermatosen (Lichen sclerosus und Lichen planus) über die schnell fortschreitende Präkanzerose differenzierte penile intraepitheliale Neoplasie (dPeIN). Diese entzündungsassoziierten, meist hoch differenzierten verhornten Karzinome enthalten Mutationen in Tumorsuppressorgenen *CDKN2A* und *TP53*. Missense-TP53-Mutationen führen zur Ansammlung von aberrantem p53-Protein in Kernen proliferierender Tumorzellen (nukleäre Überexpression). Die restlichen HPV-negativen, überwiegend verrukösen Peniskarzinome entstehen ohne Dermatosen und Tumorsuppressorgenmutationen und sind ohne p16- und p53-Überexpression. Sie entwickeln sich über verruköse PeIN. Die korrekte ätiologische Zuordnung hat klinische Bedeutung, denn HPV-assoziierte Peniskarzinome haben eine bessere Prognose und Überlebensraten. Sie ist insbesondere wichtig für die Wahl der Therapie der Präkanzerosen. Die langsam fortscheitende HSIL kann chirurgisch, destruktiv oder medikamentös über einen längeren Zeitraum behandelt werden. dPeIN dagegen sollte zeitnah chirurgisch saniert werden, um eine frühe Invasion auszuschließen. Leitliniengerechte Therapie der Dermatosen kann das Karzinomrisiko reduzieren.

## Abstract (en)

Penile squamous cell carcinoma (SSC) accounts for almost 10% of male cancers in high-incidence areas, although the incidence in Europe is below 1%. About half of penile SCCs arise from a precancerous high-grade squamous intraepithelial lesion (HSIL) caused by a transforming infection with human papillomavirus (HPV), most often high-risk HPV16. The HPV E6/E7 oncoproteins bind to proteins of the p53 and retinoblastoma pathways. This cell cycle disruption results in cellular accumulation/overexpression of p16, which serves as surrogate biomarker for HPV-associated carcinogenesis. The majority of HPV-independent SCCs arise in lesions of lichenoid dermatoses (lichen sclerosus and lichen planus) via rapidly progressing precancerous differentiated penile intraepithelial neoplasms (d-PeIN). These inflammation-associated, generally highly differentiated keratinized lesions commonly carry mutations in the tumor suppressor genes *TP53* and *CDKN2A*. Missense *TP53* mutations lead to accumulation of p53/agerrant p53 in the nuclei of proliferating tumor cells (nuclear overexpression), which serves as a surrogate marker for a *TP53* missense mutation. About one third of HPV-independent penile SCCs arise in the absence of dermatoses and mutations in tumor suppressor genes and lack p16 and p53 overexpression. They arise via verrucous/verruciform PeIN. Correct identification of the etiology of precursor lesions is of clinical significance, as HPV-associated SCCs have better prognoses and survival rates. Moreover, the etiology is particularly relevant to the choice of treatment for the precancerous lesion. The slow progression of HSIL to invasive cancers allows time-intense surgical, destructive, or drug-based treatment options. In contrast, the precursor lesion of dermatoses-associated SCC, d‑PeIN, calls for immediate surgical resection to exclude early invasion. Guideline-conform treatment of lichenoid dermatoses reduces the cancer risk.

Karzinome des Penis zählen in Europa zu den seltenen Karzinomen. Ätiologisch spielt bei etwa 50 % der Peniskarzinome eine transformierende Infektion mit humanen Papillomaviren (HPV) eine Rolle. HPV-unabhängige Peniskarzinome entstehen oft in chronisch entzündlichen Dermatosen. Die Ätiologie beeinflusst die Therapie der Präkanzerosen und die Prognose der invasiven Karzinome. Eine HPV-Impfung kann vor HPV-assoziierten Peniskarzinomen schützen. Eine leitliniengerechter Therapie der Dermatosen kann auch sowohl das Entartungsrisikos wie auch das Risiko eines Rezidivs verringern.

## Ätiologie der Peniskarzinome

Plattenepithelkarzinome des Penis zählen in den sog*. *High-incidence-Ländern Südamerikas und Afrikas mit etwa 10 % zu den häufigen Karzinomen beim Mann. In Nordeuropa sind sie sehr selten mit einer Inzidenz < 1 %. Peniskarzinome entstehen entweder nach einer persistierenden und transformierenden Infektion mit HPV-high-risk-Genotypen [1] oder unabhängig von HPV [2].

Die HPV-assoziierte Karzinogenese ist organübergreifend seit Jahrzehnten gut erforscht und kann durch Impfung verhindert werden. Die HPV-unabhängige Karzinogenese ist dagegen kaum erforscht. Chronisch-entzündliche Dermatosen spielen aber in der Entstehung eine wichtige Rolle [3]. Unabhängig von der Ätiologie stellen invasive und insbesondere metastasierte Peniskarzinome eine therapeutische Herausforderung dar. Bisher konnten keine Peniskarzinom-spezifischen Mutationen identifiziert werden, und nur in einem geringen Prozentsatz liegen sog. „Druggable“-Mutationen vor ([4, 5]; Details s. Tab. 1).

**Tab. 1:** Penile Karzinogenese

	HPV-assoziiert	HPV-unabhängig/negativ
HPV-Subtypen	HPV 16 > 31, 52, 56, 18, 45	Keine
Risikofaktoren	Zahlreiche Sexualpartner	Chronische Dermatosen, Lichen sclerosus: verhornte Haut, Lichen planus: in verhornter Haut, Schleimhäuten/Urethra
	Immunsuppression	
	HIV	
	Rauchen	
Histologien invasiver Karzinome	Unverhornt/basaloid	Hochdifferenziert/verhornt
	Klarzellig	Verrukös/verruciform
	Kondylomatös	Sarkomatoid
	Lymphoepithelial	Basaloid
Spezifische pathogene Mutationen	**–**	In Tumorsuppressorgenen *TP53 und CDKN2A in dermatosenassoziierten Karzinomen *
		HRAS-Mutationen
Bio/Surrogatmarker	*p16-Überexpression* durch E6/E7-Onkogen vermittelte Zellzyklusstörung	*Nukleäre p53-Überexpression* bei Missense-*TP53*-Genmutationen „*null pattern***“** bei disruptiven *TP53-*Mutationen
Präkanzerosen/Karzinoma in situ	Penile hochgradige squamöse intraepitheliale Läsion (*HSIL*)	Differenzierte penile intraepitheliale Neoplasie (*dPeIN*); assoziiert mit Lichen planus und Lichen sclerosus
	–	Verruköse/verruciforme penile intraepitheliale Neoplasie (vPeIN)
Klinischer Verlauf	Langsame Entwicklung von HSIL und Invasion über Jahre und Jahrzehnte	*Schnelle*, oft multifokale *Entwicklung *von *dPeIN *in Läsionen von Lichen planus und Lichen sclerosus:
	–	*Langsame Entwicklung* solitärer *verruköser Präkanzerosen* und indolenter verruköser Karzinome
Therapie der Präkanzerosen	Vielfältige Therapieoptionen: Chirurgisch, destruktiv (Kryo, Laser), zeitintensiv medikamentös (z. B. „off-label use Imiquimod“), Lebensstilmodifikation (Rauchen!!)	Zeitnahe chirurgische Exzision; keine Indikation für zeitintensive medikamentöse Therapie
Prognose	Längere Überlebenszeit	Dermatosenassoziierte Karzinome: kürzere Überlebenszeit; verruköse Karzinome: indolenter Verlauf
Prävention	HPV-Impfung	Leitliniengerechte Therapie der Dermatosen, engmaschige Kontrolle

*HPV* humane Papillomaviren, *HIV* humaner Immundefizienzvirus

### HPV-assoziierte Peniskarzinome

Im Laufe des Lebens kommt jeder sexuell aktive Mensch mit HPV in Kontakt. Risikofaktoren für die Karzinomentstehung sind häufig wechselnde Sexualpartner und ein geschwächtes Immunsystem oder Immunsuppression (z. B. Organtransplantationen oder HIV-Infektion). Zudem wird Rauchen beim Fortschreiten von Präkanzerosen zu invasiven Karzinomen impliziert. Der häufigste HPV-Subtyp in Peniskarzinomen ist High-risk-HPV 16, gefolgt von HPV 31, 52, 56, 18, 45. Nur selten entstehen kondylomatöse Peniskarzinome aufgrund von Low-risk-HPV-6- und 11-Infektionen (sog. Riesenkondylome nach Buschke u. Löwenstein; [6]).

Die HPV-assoziierten Karzinome entwickeln sich langsam über Jahre und Jahrzehnte nach der Erstinfektion, die fast immer unbemerkt, also subklinisch verläuft. Manche Patienten entwickeln aber eine sog. *produktive Infektion* mit sichtbaren Läsionen eines Condyloma accuminatum, einer Erythroplakie (Querat) oder einer Leukoplakie. In diesem Stadium kann ein intaktes Immunsystem die HPV-Infektion bekämpfen.

HPV-assoziierte Karzinome entwickeln sich langsam

Nach Rückbildung der Läsionen spricht man von *Regression*. Klinisch unbemerkt bleiben allerdings einige wenige HPV in basalen Stammzellen des Plattenepithels erhalten. In diesem sog. *Latenzstadium* liegt die Anzahl der HPV dann unterhalb der Nachweisgrenze der klinischen HPV-Tests (was klinisch oft als *HPV-Negativierung* bezeichnet wird). Je nach Immunlage kann diese Infektion oft erst Jahre/Jahrzehnte später wieder aktiv werden.

Kommt es nach einer länger persistierenden produktiven (primären oder auch reaktivierten) Infektion zur malignen Transformation, entwickelt sich zuerst eine autonom proliferierende intraepitheliale (nicht invasive) Präkanzerose mit basaloider (niedrig differenzierter) Histologie. Die HPV-assoziierten Präkanzerosen nach WHO werden als *hochgradige squamöse intraepitheliale Läsion* (HSIL) bezeichnet [1].

**Abb. 1:**
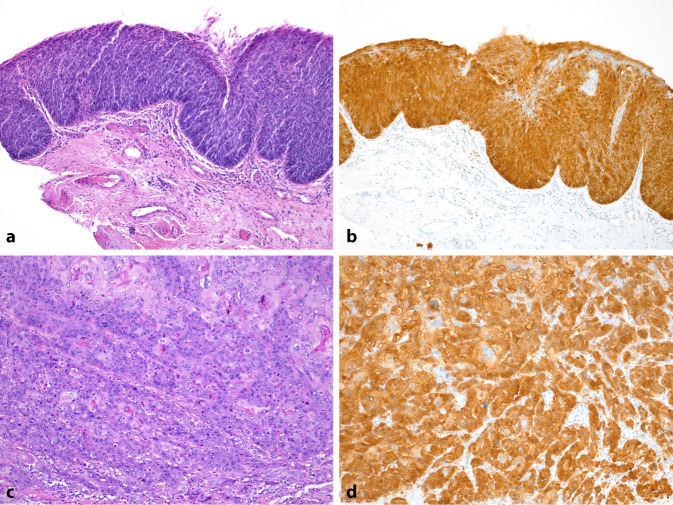
HPV-assoziierte (humane Papillomaviren) Präkanzerose und invasives Peniskarzinom: **a** HE-Färbung (Hämatoxilin-Eosin) einer HSIL (hochgradige squamöse intraepitheliale Läsion). Es zeigt sich eine die ganze Epitheldicke umfassende dysplastische Proliferation ohne Durchbruch durch die Basalmembran. **b** p16-Überexpression in allen dysplastischen undifferenzierten basaloiden Zellen der HSIL („block-like staining“). **c** HE-Färbung eines niedrig differenzierten invasiven Karzinoms. **d** p16-Überexpression in allen invasiven Zellen des Karzinoms. (Vergrößerung: **a**,**b** 10x und **c**,**d** 20x)

Die karzinogene Wirkung von HPV entfaltet sich durch Bindung der E6- und E7-Onkoproteine an Proteine der p53- und Retinoblastom-Zellzyklus-Signalwege. Als Folge dieser Blockade kommt es zur intrazellulären Ansammlung von p16. Diese kann im Tumorgewebe immunhistochemisch nachgewiesen werden (Abb. 1).

Eine einheitliche starke „Block-like“-Anfärbung von p16 wird als Überexpression definiert und gilt als indirekter Nachweis und zuverlässiger Biomarker für eine HPV-induzierte Karzinogenese [7]. Derzeit wird angenommen, dass die E6/E7-vermittelte Zellzyklusstörung für die Karzinogenese ausreicht, denn pathogene somatische Genmutationen treten erst im invasiven Stadium in < 25 % der Karzinome auf [5].

### HPV-unabhängige Plattenepithelkarzinome

Die HPV-unabhängigen Plattenepithelkarzinome zeigen keine p16-Überexpression. Etwa zwei Drittel der HPV-unabhängigen Plattenepithelkarzinome im Anogenitalbereich (Penis und Vulva) sind mit chronischen, meist unbehandelten entzündlichen Dermatosen *Lichen planus* (LP) und *Lichen sclerosus* (LS) assoziiert. Peniskarzinome entstehen in Läsionen von LS und LP an der Innenseite des Präputiums, im Sulcus coronarius, aber auch an der Glans penis. Der LP (aber nicht der LS) manifestiert sich auch in Schleimhäuten (z. B. Mundhöhle) und ist mit einem erhöhten Risiko für Karzinomentwicklung assoziiert [8, 9]. Daher sollten LP-Läsionen in der Schleimhaut des Meatus urethrae und in der Urethra besonders gründlich kontrolliert werden.

Der LS und LP enden früher oder später in entzündlichen Phimosen. In den Zirkumzisionspräparaten werden dann gelegentlich zufällig Präkanzerosen und frühinvasive Karzinome entdeckt. Typisch für dermatosenassoziierte Plattenepithelkarzinome sind Mutationen in Tumorsuppressorgenen *TP53* [10] und *CDKN2A *[5]. *TP53-*Mutationen beeinträchtigen die Bildung des p53-Proteins und resultieren in einem aberranten Expressionsmuster von p53 in immunhistochemischen Untersuchungen.

LS und LP enden früher oder später in entzündlichen Phimosen

In nicht-mutierten Karzinomen liegt ein sog. *Wildtypmuster* mit einzelnen positiven p53-Zellkernen vor. Bei Missense-Mutationen wird ein aberrantes p53 gebildet, das sich in den Kernen der teilungsfähigen Karzinomzellen ansammelt. Dies wird als nukleäre p53-Überexpression bezeichnet [3, 10]. Bei Nonsense-Mutationen wird kein p53-Protein gebildet, und die immunhistochemische Färbung ist komplett negativ (sog. *Null-Muster*). Das *CDKN2A*-Gen kodiert für die Proteine p16 und p14. Die meisten *CDKN2A*-Mutationen sind disruptiv, so dass kein p16 gebildet wird. Die seltenen *CDKN2A*-missense-Mutationen dagegen führen zur Bildung eines aberranten p16, daher kann eine p16-Färbung beobachtet werden [11].

Ein kleiner Anteil an HPV-negativen, p16-negativen Karzinome entsteht ohne lichenoide Dermatosen und ohne Mutationen in Tumorsuppressorgenen. Sie zeigen ein p53-Wildtypmuster und Genmutationen in *PIK3CA, FGFR‑3* und *HRAS* [5]. Histologisch handelt es sich um verhornte verruköse Karzinome mit breiter plumper Invasionsfront oder in Schleimhautnähe um unverhornte papilläre Karzinome. Diese Karzinome haben einen indolenten Verlauf und kein Metastasierungspotential.

## Histologie der invasiven Peniskarzinome

In den letzten Jahrzehnten wurde eine verwirrende Vielzahl von beschreibenden, schlecht reproduzierbaren histologischen Diagnosen in der Diagnostik verwendet. Diese stammten überwiegend aus der Prä-HPV-Ära. Erst die Einführung der Genotypisierung und klinischen HPV-Testungen erlaubten eine ätiologische Zuordnung der Peniskarzinome. Mit Hilfe des kostengünstigen Surrogatmarkers p16 wurde die histologische Klassifikation in den letzten 15 Jahren deutlich vereinfacht und verschlankt [7]. In der aktuellen *WHO Classification of Urological Tumors* (2022; [1]) werden für HPV-assoziierte Karzinome nur mehr die histologischen Subtypen basaloid, klarzellig [12], lymphoepithelial und kondylomatös beschrieben.

Die meisten HPV-unabhängigen Karzinome sind hochdifferenziert und verhornt [2, 3, 5, 10]. Sie zeigen oft ein charakteristisches Invasionsmuster mit Einzelzellinfiltraten und Zellsträngen [13]. Diese Einzelzellen können schnell über die Lymphgefäße in die regionalen Lymphknoten metastasieren. Die verrukösen und papillären/verruciform HPV-negative Karzinome haben breite plumpe Invasionsfronten. Selten ist eine sarkomatoide Differenzierung nachweisbar.

## TNM-Klassifikation der Peniskarzinome

Unabhängig von der Ätiologie werden invasive Peniskarzinome nach TNM („tumour, node, metastasis“) klassifiziert (letzte Überarbeitung 2017; [14]). Die primären Karzinome mit Invasion in das subepitheliale Bindegewebe werden als pT1-Karzinome klassifiziert. Je nach histologischem Differenzierungsgrad und Nachweis von Lymphgefäßinvasion werden sie noch zusätzlich in pT1a (histologischer Grad 1 und 2, keine Gefäßinvasion) und pT1b (histologischer Grad 3 und/oder Gefäßinvasion) unterteilt. Der Prozentsatz an pT1a-Karzinomen ist bei den HPV-unabhängigen Tumoren etwas höher als bei den HPV-assoziierten Karzinomen. Dies ist wahrscheinlich dem schnellen Fortschreiten der HPV-unabhängigen Präkanzerosen und der zufälligen Entdeckung von frühinvasiven Karzinomen in Zirkumzisionspräparaten von Phimosen geschuldet [5].

Die derzeitige Datenlage spricht dafür, dass pT1a-Karzinome beider Ätiologien kein metastatisches Potential haben. Ab dem pT1b-Stadium liegen ausgedehntere invasive Tumoren vor, die auch das Potential zur lymphogenen Invasion haben [15]. Die histopathologische Aufarbeitung der Sentinel-Lymphknoten mit Serienschnittstufen (Hämatoxilin-Eosin-Färbungen und immunhistochemischen Untersuchungen mit Antikörper gegen Zytokeratin) hilft in der Beurteilung der frühen regionalen Metastasierung bei insgesamt geringer Morbidität [16]. Sie erlaubt den Nachweis einzelner therapierelevanter metastatischer Karzinomzellen. In Analogie zum Vulvakarzinom [17] wird die Einzelmetastasierung überwiegend bei HPV-unabhängigen Peniskarzinomen erwartet.

## Präkanzerosen der Peniskarzinome

Aufgrund der begrenzten Therapiemöglichkeiten größerer invasiver Peniskarzinome ist es wichtig, die präinvasiven Stadien rechtzeitig zu erkennen und adäquat zu therapieren. In Analogie zu anderen HPV-assoziierten anogenitalen Plattenepithelkarzinomen bezeichnet die WHO die HPV-assoziierten penilen Präkanzerosen als HSIL. Die undifferenzierte Proliferation durchsetzt die gesamte Epitheldicke und ist durch eine starke, einheitliche p16-Anfärbung charakterisiert (Abb. 1).

Die HPV-unabhängigen Präkanzerosen werden in dPeIN und vPeIN unterteilt

Die HPV-unabhängigen Präkanzerosen werden – ebenfalls in Analogie zu HPV-unabhängigen Präkanzerosen an der Vulva und Zervix – in differenzierte penile intraepitheliale Neoplasie (dPeIN) und verruköse/verruciforme penile intraepitheliale Neoplasie (vPeIN) unterteilt [18]. Sie entstehen oft multifokal in Läsionen von LP und LS.

**Abb. 2:**
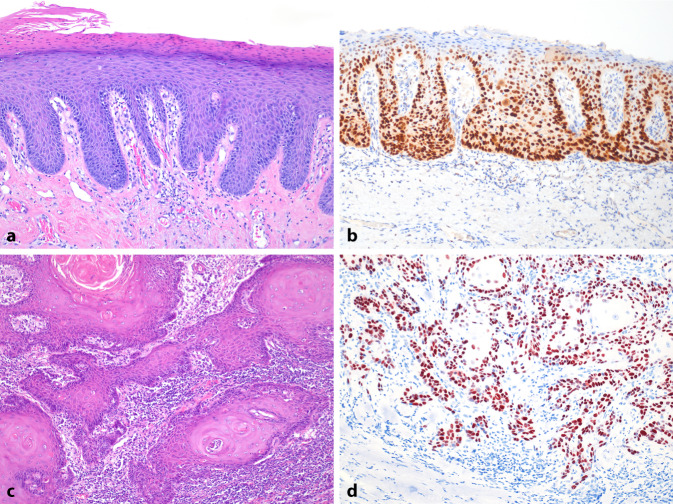
Humane Papillomavirus unabhängige Präkanzerose und invasives Peniskarzinom: **a** Hämatoxilin-Eosin-Färbung einer differenzierten penilen intraepithelialen Neoplasie (dPeIN) mit deutlich verlängerten Reteleisten, Para- und Hyperkeratose; **b** mit nukleärer p53-Überexpression in den basalen und suprabasalen Zellen der verlängerten Reteleistungen der dPeIN. **c** HE-Färbung eines hoch differenzierten verhornten invasiven Karzinoms mit. **d** Nukleärer p53-Überexpression in den invasiven Zellen des Karzinoms. (Vergrößerung: **a**-**c** 10x, **d** 20x)

Charakteristisch für die dPeIN sind eine atypische basalzellige Proliferation mit nukleärer p53-Überexpression in verlängerten Reteleisten und einer abrupten vorzeitigen Verhornung (Abb. 2). Die oberflächliche Verhornung des Plattenepithels kann normal sein, allerdings zeigen viele dPeIN eine schuppige Oberfläche mit Parakeratose. Die verhornten verrukösen und unverhornten verruciformen PeIN sind meist solitäre hochdifferenzierte Läsionen mit minimalen Zellatypien ohne nennenswerte mitotische Aktivität.

Die ätiologische Unterteilung der Präkanzerosen basierend auf dem HPV-Status hat wichtige therapeutische Konsequenzen. Die HPV-assoziierte Karzinogenese ist ein langsamer Prozess. Daher können für die HSIL verschiedene Therapiekonzepte angeboten werden; neben chirurgischer Intervention und Destruktion können auch eine zeitintensive topische medikamentöse Behandlung (z. B. mit Imiquimod [„off-label use“] über mehrere Monate) zum Einsatz kommen. Auch eine Lebensstiländerung (z. B. Raucherentwöhnung) kann die Therapie unterstützen.

Auch die HPV-unabhängigen verrukösen/verruciformen PeIN sind in ihrem Verlauf langsam und indolent. Die HPV-unabhängige dPeIN dagegen ist als schnellfortschreitende Präkanzerose zu werten, die trotz oberflächlicher Ausreifung und hoch differenzierter Histologie schnell invasiv werden kann.

## Risikofaktoren HPV und lichenoide Dermatosen

Ein wichtiger Fokus liegt auf der Prävention von Präkanzerosen und invasiven Peniskarzinomen. Eine rechtzeitige HPV-Impfung im Schulalter kann HPV-assoziierte Karzinome deutlich reduzieren, wenn nicht gar verhindern. Leider gibt es keine kausale Prävention für die HPV-unabhängige Karzinogenese. LS und LP sind im Spektrum der Immundysregulierung/Autoimmunerkrankungen bei genetischer Disposition einzureihen. Eine leitliniengerechte Therapie dieser Dermatosen kann die Morbidität und auch das Risiko der Entartung verringern [19].

Regelmäßige und engmaschige Kontrollen und klinische Kenntnis der mannigfaltigen Präsentationen von LP sind für eine frühzeitige Diagnose von Präkanzerosen hilfreich. Klinisch präsentiert sich der LS mit größeren, glatten, weißlichen Plaques in verhornter Haut. Der LP dagegen hat mehrere klinische Präsentationen. Typisch in verhornter Haut sind gruppierte kleine Papeln mit abgeflachter „planer“ Oberfläche. Bei deutlicher Verhornung der Papeln spricht man vom hypertrophen LP. Hier stellt sich klinisch oft die Differentialdiagnose einer verrukösen Präkanzerose.

In größerflächigen Läsionen entstehen Präkanzerosen oft multifokal. Alternativ manifestiert sich der LP mit glänzenden, scharf begrenzten Erythemen, die von verdickter weißlicher Schleimhaut (sog. Wickham-Streifung oder „Wickham striae“) umgeben sind. Diese Läsionen werden auch als sog. *erosiver LP* und in der Dermatologie oft als *Lichen ruber* bezeichnet. Typische Lokalisationen sind an der Glans penis und der Innenseite der Vorhaut. Besonders wichtig ist die Inspektion des Meatus urethrae und der distalen Harnröhre. Nach einer Zirkumzision sollte eine topische Therapie der residuellen Dermatosen erfolgen.

In größerflächigen Läsionen von LP entstehen Präkanzerosen oft multifokal

Auch wenn für Peniskarzinome nur begrenzte Erfahrungswerte vorliegen, können viele Erfahrungen von vulvären Dermatosen und der vulvären Karzinogenese auf den Penis übertragen werden. Der LS ist die unkompliziertere Dermatose, mit häufiger kompletter Remission, während der LP schwieriger zu therapieren ist. Die Lokaltherapie kann in Analogie zur Vulva primär mit hochpotenten Kortikosteroiden für bis zu 3 Monate begonnen werden [19]. Die therapieinduzierte Reduktion des Entzündungsinfiltrates kann nicht nur die Morbidität, sondern auch das Entartungsrisiko verringern [20].

## Fazit für die Praxis

- Die ätiologische Zuordnung von Peniskarzinomen und Präkanzerosen ist aus prognostischen und therapeutischen Gründen wichtig:
- Humane Papillomaviren assoziierte Peniskarzinome entstehen über die Präkanzerose hochgradige squamöse intraepitheliale Läsion (HSIL). p16 gilt als Surrogat-Marker für die HPV-Ätiologie und korreliert mit besserer Prognose.
- Die langsame Progression von HSIL erlaubt chirurgische und destruktive, aber auch zeitintensive medikamentöse Therapien
- Die meisten HPV-unabhängigen, p16-negativen Karzinome sind mit Lichen planus (LP) oder Lichen sclerosus (LS) assoziiert. Sie enthalten Tumorsuppressorgenmutationen und entstehen über die schnell fortschreitende Präkanzerose differenzierte penile intraepitheliale Neoplasie (dPeIN). Eine *TP53*-missense-Mutation führt zu nukleärer p53-Überexpression.
- dPeIN erfordern eine zeitnahe chirurgische Exzision, um eine frühe Invasion auszuschließen.
- Eine Therapie der Dermatosen kann das Krebsrisiko verringern.
- Verruköse Präkanzerosen und Karzinome ohne Dermatosen haben einen indolenten Verlauf.

## References

[1] Alvarado-Cabrero I, Chaux A, Muneer A et al (2022) HPV-associated squamous cell carcinoma, in WHO Classification of Tumors. Urinary and male genital tumours. IARC, Lyon, France, S 372–374

[2] Alvarado-Cabrero I, Canete-Portillo S, Chaux A et al (2022) HPV-independent squamous cell carcinoma, in WHO Classification of Tumours. IARC, Lyon, France, S 375–379

[3] Mannweiler S, Sygulla S, Winter E et al (2013) Two major pathways of penile carcinogenesis: HPV-induced penile cancers overexpress p16, HPV-negative cancers associated with dermatoses express p53, but lack p16 overexpression. J Am Acad Dermatol 69(1):73–81 23474228 10.1016/j.jaad.2012.12.973

[4] Chahoud J, Pham R, Sonpavde G (2022) Innovative systemic therapies for penile cancer. Curr Opin Urol 32(1):8–16 34738984 10.1097/MOU.0000000000000941

[5] Ermakov MS, Kashofer K, Regauer S (2023) Different Mutational Landscape in HPV-induced and HPV-independent Invasive Penile Squamous Cell Cancers. Mod Pathol: 100250 (p) 10.1016/j.modpat.2023.100250 37353203

[6] Mannweiler S, Sygulla S, Beham-Schmid C et al (2011) Penile carcinogenesis in a low-incidence area: a clinicopathologic and molecular analysis of 115 invasive carcinomas with special emphasis on chronic inflammatory skin diseases. Am J Surg Pathol 35(7):998–1006 21681144 10.1097/PAS.0b013e3182147e59

[7] Mustasam A, Parza K, Ionescu F et al (2025) The Prognostic Role of HPV or p16INK4a Status in Penile Squamous Cell Carcinoma: A Meta-Analysis. J Natl Compr Canc Netw 23(2):e247078 10.6004/jnccn.2024.7078 39752880

[8] Gandolfo S, Richiardi L, Carrozzo M et al (2004) Risk of oral squamous cell carcinoma in 402 patients with oral lichen planus: a follow-up study in an Italian population. Oral Oncol 40(1):77–83 14662419 10.1016/s1368-8375(03)00139-8

[9] Chryssostalis A, Gaudric M, Terris B et al (2008) Esophageal lichen planus: a series of eight cases including a patient with esophageal verrucous carcinoma. A case series. Endoscopy 40(9):764–768 18535938 10.1055/s-2008-1077357

[10] Kashofer K, Winter E, Halbwedl I et al (2017) HPV-negative penile squamous cell carcinoma: disruptive mutations in the TP53 gene are common. Mod Pathol 30(7):1013–1020 28387325 10.1038/modpathol.2017.26

[11] May M, Hrudka J, Elst L et al (2025) Letter to the Editor: Advancing Prognostic Stratification in PSCC: Developing Predictive Models as the Next Pivotal Step. J Natl Compr Canc Netw 23(5) 10.6004/jnccn.2025.7038 40341136

[12] Liegl B, Regauer S (2004) Penile clear cell carcinoma: a report of 5 cases of a distinct entity. Am J Surg Pathol 28(11):1513–1517 15489656 10.1097/01.pas.0000141405.64462.2a

[13] Hrudka J, Prouzova Z, Bartu MK et al (2023) Immune cell infiltration, tumour budding, and the p53 expression pattern are important predictors in penile squamous cell carcinoma: a retrospective study of 152 cases. Pathology 55(5):637–649 37316384 10.1016/j.pathol.2023.03.010

[14] UICC (Hrsg) (2017) TNM Classification of Malignant Tumours, 8. Aufl. John Wiley & Sons, Oxford, UK

[15] Mannweiler S, Sygulla S, Tsybrovskyy O et al (2012) Clear-cell differentiation and lymphatic invasion, but not the revised TNM classification, predict lymph node metastases in pT1 penile cancer: a clinicopathologic study of 76 patients from a low incidence area. Urol Oncol 31(7):1378–1385 22421354 10.1016/j.urolonc.2012.01.017

[16] Elversang J, Gronkaer Toft B, Predbjorn Krarup K et al (2021) Lessons learned from histological step-sectioning of sentinel lymph nodes in penile cancer. Histopathology 78(4):627–633 32979281 10.1111/his.14261

[17] Regauer S (2009) Histopathological work-up and interpretation of sentinel lymph nodes removed for vulvar squamous cell carcinoma. Histopathology 55(2):174–181 19694824 10.1111/j.1365-2559.2009.03350.x

[18] Regauer S, Ermakov M, Kashofer K (2023) The Spectrum of HPV-independent Penile Intraepithelial Neoplasia: A Proposal for Subclassification. Am J Surg Pathol 47(12):1449–1460 37768009 10.1097/PAS.0000000000002130 PMC10642695

[19] Kirtschig G, Kinberger M, Kreuter A et al (2024) EuroGuiderm guideline on lichen sclerosus-introduction into lichen sclerosus. J Eur Acad Dermatol Venereol 38(10):1850–1873 38822578 10.1111/jdv.20082

[20] Cooper SM, Madnani N, Margesson L (2015) Reduced Risk of Squamous Cell Carcinoma With Adequate Treatment of Vulvar Lichen Sclerosus. JAMA Dermatol 151(10):1059–1060 26069940 10.1001/jamadermatol.2015.0644

